# Channel Code-Book (CCB): Semantic Image-Adaptive Transmission in Satellite–Ground Scenario

**DOI:** 10.3390/s25010269

**Published:** 2025-01-06

**Authors:** Hui Cao, Shujun Han, Rui Meng, Xiaodong Xu, Ping Zhang

**Affiliations:** State Key Laboratory of Networking and Switching Technology, Beijing University of Posts and Telecommunications, Beijing 100876, China; caohui@bupt.edu.cn (H.C.); hanshujun@bupt.edu.cn (S.H.); buptmengrui@bupt.edu.cn (R.M.); pzhang@bupt.edu.cn (P.Z.)

**Keywords:** semantic communication, satellite–ground scenarios, channel adaptive, image transmission

## Abstract

Satellite–ground communication is a critical component in the global communication system, significantly contributing to environmental monitoring, radio and television broadcasting, aerospace operations, and other domains. However, the technology encounters challenges in data transmission efficiency, due to the drastic alterations in the communication channel caused by the rapid movement of satellites. In comparison to traditional transmission methods, semantic communication (SemCom) technology enhances transmission efficiency by comprehending and leveraging the intrinsic meaning of information, making it ideal for image transmission in satellite communications. Nevertheless, current SemCom methods still struggle to adapt to varying channel conditions. To address this, we propose a SemCom transmission model based on a Channel Code-Book (CCB) for adaptive image transmission in diverse channel environments. Our model reconstructs and restores the original image by documenting fading and noise states under various channel conditions and dynamically adjusting the denoiser’s model parameters. Extensive experimental results demonstrate that our CCB model outperforms three representative baseline models, including Deep JSCC, ASCN, and WITT in various environments and task conditions, achieving an advantage of more than 10 dB under high signal-to-noise ratio conditions.

## 1. Introduction

### 1.1. Background

Since its inception in the mid-20th century, satellite communication has emerged as a vital component in the global communication system [1]. Its wide coverage, high reliability, and adaptability to various geographical environments have made it indispensable in various fields, including military operations, meteorology, navigation, broadcasting, and the Internet [2]. With the relentless progression of science and technology, modern satellite communication systems have witnessed remarkable enhancements in data transmission rates, spectrum utilization efficiency, and anti-interference capabilities. In recent years, the deployment and utilization of low Earth orbit (LEO) satellites have facilitated faster and lower-latency communication services worldwide, further accelerating the development and application of satellite communication technology. These technologies will play a pivotal role in the development of 6G and other networks that integrate new functions such as sensing and computing, allow the use of new services, and leverage improved environmental information for machine learning (ML) and artificial intelligence (AI) [3,4,5].

However, satellite–ground communication currently faces numerous challenges in the process of image transmission. These challenges primarily involve environmental interference, spectrum resources, and energy management [6]. Firstly, as the signal traverses the atmosphere, it is subjected to atmospheric attenuation and meteorological conditions, resulting in path loss and signal degradation. Concurrently, multipath effects cause signal interference, further diminishing transmission quality, increasing bit error rates, and reducing the accuracy and integrity of the transmission [7]. Additionally, limited spectrum resources constrain the transmission bandwidth and rate of image data. Furthermore, the finite energy resources of satellites necessitate a careful balance between transmission power and energy consumption to ensure the normal operation and longevity of the satellite [8].

### 1.2. SemCom-Enabled Satellite Communications

To achieve stable satellite-to-ground image transmission, scholars have conducted extensive research on image restoration. In [9], the dark channel prior method is employed to enhance the visual clarity of satellite photographs and reduce haze, particularly in poor weather conditions. Yang et al. in [10], utilized techniques such as multi-band digital image color synthesis and multi-spectral image band combination to further improve the clarity of satellite images. Additionally, the study presented in [11] applies a recursive least squares (RLS) adaptive filter to enhance the recovery performance of retrieved satellite images. However, these methods require a large amount of real-time computing, which puts forward huge requirements for satellite computing power and cannot meet the growing demand for data transmission.

In this scenario, the burgeoning Semantic Communication (SemCom) technology has increasingly come into focus. The SemCom system leverages logical reasoning capabilities to grasp the inherent meaning of information [12], specifically the intent of the message, and maps the signal space onto the semantic space. This approach significantly diminishes the volume of business transmission and substantially enhances transmission efficiency [13]. SemCom goes beyond mere data transmission, placing equal emphasis on the semantic information underlying the data [14]. By extracting and utilizing this semantic information, it facilitates more efficient transmission within limited bandwidth constraints [15]. Consequently, SemCom is exceptionally well-suited for the extensive multimedia data transmissions involved in satellite communications, including images and videos [16].

Recent studies have explored the application of SemCom in satellite–ground scenarios from various perspectives. Regarding frequency band selection, Zhu et al. [17] proposed a multi-agent reinforcement learning method that significantly enhances classification accuracy. For energy efficiency optimization, the integration of a privacy-preserving task-offloading algorithm [18] and an ML-based semantic encoder [19] has achieved a balance between task completion time, energy consumption, and the quality of service. Furthermore, the scope and efficiency of task processing have been enhanced through the implementation of a non-ground network architecture [20] and a task-offloading framework [21].

In the realm of routing optimization, Guo et al. [22] developed a SemCom-aware routing scheme to improve data throughput and end-to-end delay performance. Another study [23] enhanced transmission efficiency by leveraging time-varying channel conditions and specific content through a routing-based semantic adaptive coding and hybrid automatic repeat request (HARQ) mechanism. Additionally, the semantic enhancement system uses orthogonal time-frequency space (OTFS) modulation and generative adversarial network (GAN) to extract and recover semantic features, which significantly improves signal-to-noise ratio (SNR) gain and spectral efficiency [24].

For information perception, an adaptive semantic encoder–decoder architecture focuses on utilizing channel conditions and content correlation to protect and restore specific functions [25]. The perception–communication–computing–actuation–integrated paradigm (PCCAIP), as proposed in [26], optimizes communication process through the integration of sensing, computing, and actuation technologies. In terms of delay optimization, papers [24,25,27] have improved transmission efficiency by integrating SemCom with different modulation methods.

### 1.3. Motivations and Contributions

While these research results provide significant theoretical and technical support for enhancing the efficiency and reliability of satellite–ground communication systems, several issues remain in the image transmission algorithm within the satellite–ground scenario using SemCom. Firstly, unlike traditional ground networks, LEO satellites are in constant high-speed motion, resulting in a continuously changing communication environment with the ground. The fast fluctuating channel is characterized by significant changes in channel parameters over short periods, making it challenging for traditional satellite–ground image transmission models to adapt in real time, resulting in a decline in transmission performance. Additionally, fast fluctuation channels are typically accompanied by multipath fading, which causes signal interference and jitter, negatively impacting the stability and quality of image transmission. Furthermore, the spectrum compression effect is less pronounced in fast fluctuation channels compared to stable channels, thereby limiting the effectiveness of satellite–ground image transmission models in high-data-rate scenarios. This dynamic environment necessitates that the image transmission algorithm using SemCom consider more samples of different states during model training. Particularly for transmission algorithms employing pre-trained models, discrepancies between actual channel conditions and training conditions can lead to a rapid decline in performance. Although current iterative decoding methods [28,29,30] can accurately correct errors and improve the quality of transmitted information by combining semantic and syntactic information, they require multiple calculations. This significantly increases computational complexity and time overhead, rendering them unsuitable for deployment in satellite–ground scenarios where computing power and delay are limited. Secondly, current SemCom algorithms based on deep learning are typically designed and trained with a joint encoder–decoder configuration. Consequently, any changes in the decoder configuration require corresponding adjustments in the encoder configuration. However, in satellite–ground transmission, a satellite often needs to serve multiple users simultaneously. Constantly modifying the encoder configuration on the satellite side for users in different regions is impractical. Thus, it is imperative to develop a channel-adaptive transmission model that does not necessitate modifications to the satellite-side configuration.

Against this background, we propose an adaptive transmission architecture based on Channel Code-Book (CCB). The proposed framework records different channel information in the code-book in the form of parameters, obtains the current actual environmental information through the pilot signal, matches and denoises the information stored in the code-book, and finally realizes the data restoration. The main contributions of this paper are listed as follows:

- Addressing the issue of poor adaptability in image transmission models due to rapid channel changes in satellite–ground environments, we propose a SemCom framework equipped with the CCB. The framework enables reliable image transmission in various complex environments;
- To tackle the problem of signal distortion resulting from channel fading and noise, we design an Adaptive Channel Denoising Model (ACDM) as a denoising solution. This model can adaptively choose the appropriate denoising parameters based on different signal characteristics, effectively restoring faded and noisy images in satellite–ground channel environments;
- To enable the denoising model to select suitable parameters based on the current channel environment, we propose a pilot-based method for channel model parameter selection. This method can analyze the characteristics of the current channel environment and intelligently select the most appropriate parameter configuration to optimize the performance of the CCB model;
- To demonstrate the superiority of our proposed scheme, we conducted extensive simulation experiments and compared our results with those of other schemes. The simulation results indicate that, compared to the existing fixed-structure SemCom system, CCB significantly enhances the image transmission performance of the satellite–ground channels and shows stronger adaptability and stability.

The remainder of this paper is organized as follows. In Section 2, we first introduce the system model, including the network model, channel model, and the proposed CCB architecture. Then, in Section 3, we introduce the CCB MappingNet, including the design principle and training algorithm. Section 4 is dedicated to the details of the denoiser and parameter selection mechanism. Section 5 provides the experiment results and a direct comparison of several methods to quantify the performance gain of the proposed method. Finally, Section 6 concludes this paper.

## 2. System Model

This section introduces the network model, channel model, and proposed end-to-end semantic image adaptive transmission framework for satellite–ground communications.

### 2.1. Network Model

**Figure 1:**
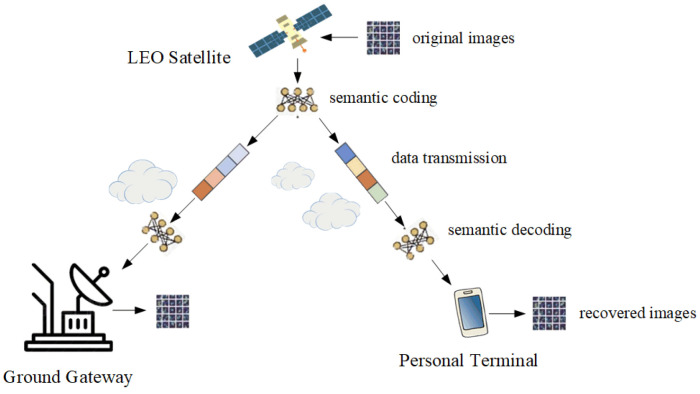
The system model of satellite–terrestrial transmission network.

We consider a scenario where images are transmitted from satellite to ground, as shown in Figure 1. The components involved are as follows:

- LEO satellite: The satellite revolves around the Earth, constantly moving and covering different regions. The satellite uses the images generated by observing the ground data as the source, and encodes these data, including compression and semantic information extraction, to improve transmission efficiency and data quality. It is necessary to consider the dynamic adjustment of the change in track and coverage areas to ensure the stability of data transmission;
- Transmission link: Links between satellites and ground equipment via wireless channels can be affected by environmental factors such as clouds and electromagnetic interference, which can degrade signal quality. In addition, due to the movement of LEO satellites, the transmission path and conditions will change with the position of the satellite, so it is necessary to dynamically adjust the transmission strategy to maintain the stability and efficient transmission of signals;
- Ground terminal: Terminal equipment includes ground gateway station and personal terminal. The terminal device receives the signal transmitted by the satellite and decodes it to restore the original image or semantic information, and the user can view or use the received information here.

### 2.2. Channel Model

Differently from the traditional terrestrial network, in the satellite–ground transmission scenario, the channel between the satellite and the ground user is not only affected by the distance loss caused by the line-of-sight (LoS) propagation, but also by the atmospheric fading, the shadow effect caused by obstacles, and the scattering. We use the shadowed-Rician (SR) fading channel, which is widely used in both S-band and ka-band satellite channels for data transmission [31,32,33]. We assume that the satellite uses a single transmitting antenna. According to [31], the probability density function (PDF) of the SR channel gain $f_{h}\left(r\right)$ is expressed as

(1) fh(r)=2b0m2b0m+Ωm12b0exp−r2b0·F11m,1,Ωr2b0(2b0m+Ω),

where $b_{0}$, *m*, Ω denote the average power of scatter component, the Nakagami-*m* parameter, and LOS component, respectively. The term F11(a,b,c) is the first kind of confluent hypergeometric function. In particular, for the parameter *m*, when *m* is small, this indicates that there are more obstacles and shadows between the satellite and the ground, which may not meet the conditions for establishing the LoS link. When *m* tends to infinity, this indicates that the link between the satellite and the ground has almost no obstacles and blockages, which satisfies the conditions of the LoS link. In this case, the SR distribution will be downgraded to the Rayleigh distribution [33].

According to the different size of the fading, paper [31] divides the channel model into three states, which are infrequent light shadowing (referred to as light), frequent heavy shadowing (referred to as heavy), and average shadowing (referred to as average).

### 2.3. Proposed Semantic Image-Adaptive Transmission Framework

**Figure 2:**
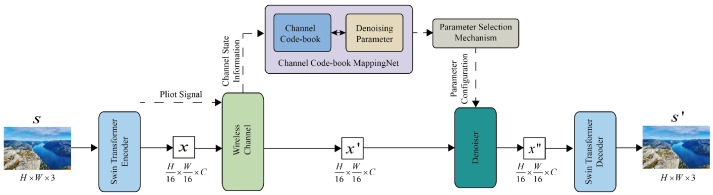
The overall architecture of CCB for satellite–ground image transmission.

An overview of the proposed CCB architecture for image transmission is presented in Figure 2. The main parts of the architecture are as follows.

#### 2.3.1. Swin Transformer Encoder

The encoder $f_{e}$ we used consists of a swin transformer encoder. The swin transformer model combines a standard multi-head self-attention (MSA) module and a feedforward network for processing [34]. The window-based self-attention mechanism allows the model to capture long-term dependencies in the image. It divides the image into window grids and applies self-attention in each window. Four cascaded swin transformer block modules are used to process high-resolution source images and learn from the changing characteristics of the transmission channel. After this step, we obtain the feature map $x\in R^{\frac{H}{16}\times \frac{W}{16}\times C}$ to represent the semantic latent representation of the input image $s\in R^{H\times W\times 3}$, where *H*, *W*, and *C* represent height, width, and channel number. The process is modeled as

(2) $$\begin{array}{c} x=f_{e}\left(s,\phi \right), \end{array}$$

where ϕ contains the model parameters corresponding to the encoder.

#### 2.3.2. Denoiser

Beyond the encoder, x is sent to the satellite–ground channel for transmission. Due to the existence of channel fading and noise, the feature vector x will change to $x^{\prime }$ after passing through the channel. The fading process is modeled as

(3) $$\begin{array}{c} x^{\prime }=hx+n, \end{array}$$

where n is random Gaussian noise, $n∼(0,{\mathrm{oe}}^{2})$, and h is the channel gain.

To reduce the error caused by noise interference in the transmission process, the denoiser is used in this architecture. The denosier adopts different model parameters in different channel environments that are trained in CCB MappingNet and chosen by the proposed parameter selection mechanism. In the denoiser, $x^{\prime }$ is transformed into $x^{\prime \prime }$, which is similar to x, and the process is modeled as

(4) $$\begin{array}{c} x^{\prime \prime }=f_{dn}\left(x^{\prime },\theta \right), \end{array}$$

where θ contains the model parameters of the denoiser.

#### 2.3.3. Swin Transformer Decoder

Finally, $x^{\prime \prime }$ is sent to the decoder. The decoder $f_{d}$ follows the symmetric architecture with the encoder $f_{e}$, including feature reconstruction, upsampling patch segmentation operation, and swin transformer. The output image $s^{\prime }$ is recovered by the decoder, and the process is represented as

(5) $$\begin{array}{c} s^{\prime }=f_{d}\left(x^{\prime \prime },\phi^{\prime }\right), \end{array}$$

where $\phi^{\prime }$ contains the model parameters of the decoder.

### 2.4. The Training Process of the End-to-End Transmission Model

For the transmission model, our goal is to minimize the difference between the output image and the input image, that is, $\left(\phi^{*},\phi^{\prime *}\right)=\arg\min_{\phi ,\phi^{\prime }}\left[d\left(s,s^{\prime }\right)\right]$, where d(·) is the loss function. We use the MSE function as the loss function. The MSE function is widely used in image restoration models [35,36,37], and the formula is

(6) $$\mathrm{MSE}=\frac{1}{n}\sum_{i=1}^{n}{\left(y_{i}-{\hat{y}}_{i}\right)}^{2},$$

where $y_{i}$ is the actual value and ${\hat{y}}_{i}$ is the predicted value. After training the end-to-end transmission model, it can be employed for real-time transmission. The whole process is outlined in Algorithm 1.
**Algorithm 1** Training and Inference of the End-to-End Transmission Model**Input:** Dateset S, train epoch $E_{p}$, learning rate $l_{r}$, batch size *b*.**Output:** Encoder parameters ϕ, decoder parameters $\phi^{\prime }$, and denoiser parameters θ.1:**Training stage**:2:Initialize the model architecture.3:**for** epoch = 1 : $E_{p}$ **do**4:    Sample a batch of images $s_{1}$, …, $s_{b}$∈S;5:    Generate semantic features $x_{1}$, …, $x_{b}$ from encoder with ϕ;6:    Transmit the bit streams to the receiver through a channel and get $x_{1}^{\prime }$, …, $x_{b}^{\prime }$;7:    Remove noise and fading effects from denoiser and get $x_{1}^{\prime \prime }$, …, $x_{b}^{\prime \prime }$;8:    Generate the images $s_{1}^{\prime }$, …, $s_{b}^{\prime }$ from decoder with $\phi^{\prime }$;9:    Calculate the loss function between $s^{\prime }$ and s;10:    Calculate the gradient and update ϕ, $\phi^{\prime }$;11:**end for**12:Set the channel environment parameters;13:**for** epoch = 1 : $E_{p}$ **do**14:    Get denoising parameters from Algorithm 2;15:    Train denoising parameters θ from Algorithm 3;16:**end for**17:**return** the model parameters ϕ, $\phi^{\prime }$, and θ.18:**Inference stage**:19:Choose denoising parameters through Algorithm 4;20:Transmit images.

**Algorithm 2** Training Algorithm for Channel Code-Book MappingNet
**Input:** Recorded channel parameters ($b_{0}$, *m*, Ω, SNR), train epoch $E_{p}$.**Output:** Denoising model parameters $\theta_{dn}$.1:Recorded fixed channel parameters ($b_{0}$, *m*, Ω, SNR).2:**for** epoch = 1 : $E_{p}$ **do**3:    Transmit x;4:    Receive the transmitted data $x^{\prime }$ through the channel with above channel parameters;5:    Generate $x^{\prime \prime }$ = $f(x^{\prime })$ with model parameter $\theta_{dn}$;6:    Calculate the loss function between $x^{\prime \prime }$ and x;7:    Calculate the gradient and update $\theta_{dn}$;8:
**end for**
9:**return** the final $\theta_{dn}$ with above channel parameters;10:Change the channel model parameters and repeat the above steps.


**Algorithm 3** Training Algorithm for Channel DenoisingNet
**Input:** Input features $x^{\prime }$.**Output:** Denoiser parameters W and b.1:Initialize the denoiser model architecture;2:Generate encoder1’s convolution kernel $W_{conv}$ and bias term $b_{conv}$ from $f_{conv}\left(x^{\prime }\right)=W_{conv}*x^{\prime }+b_{conv}$;3:Generate decoder1’s convolution kernel $W_{deconv}$ and bias term $b_{deconv}$ from $x^{\prime \prime }=f_{deconv}\left(a\right)=W_{deconv}*a+b_{deconv}$;4:Get specific $\theta_{dn}$ from Section 3;5:Transform $\theta_{dn}$ into W and b.


**Algorithm 4** Denoiser Model Parameter Selection Mechanism
**Input:** Initial transmission polit $x_{0}$, total transmission number *M*, pre-stored guiding vector $x_{n}^{\prime }$(n=1,2…N).**Output:** Denoiser parameter θ.1:Initialize denoiser parameter θ=0;2:**for** transmission number = 1 : *M* **do**3:    **if** transmission number == 1 **then**4:        Transmit $x_{0}$;5:        Receive the transmitted data $x_{n}^{\prime }$;6:        **for** Pre-stored guiding vector $x_{i}^{\prime }$ = $x_{1}^{\prime }$ : $x_{n}^{\prime }$ **do**7:           Compute the difference and set parameter: $x_{\min}^{\prime }=\mathrm{argmin}(d|x_{i}^{\prime }-x_{0}^{\prime }\left|\right)$;8:        **end for**9:        Converted into denoising parameters: $\theta ↔x_{\min}^{\prime }$;10:        Denoiser apply denoising parameters $f_{dn}=\theta$.11:    **end if**12:
**end for**



## 3. Designed Channel Code-Book MappingNet Based on Vector Quantization

In this section, we present the CCB MappingNet for image transmission under satellite–ground conditions. Firstly, we introduce the design principle of CCB. Then, we propose a channel information conversion module CCB MappingNet. Finally, we give the training algorithm of CCB MappingNet.

### 3.1. Channel Code-Book

In the satellite–ground communication environment, with the change in satellite position, the transmission channel between satellite and ground also changes greatly, so it cannot be expressed by a single channel function. Therefore, we design a CCB to store different channel parameters.

The proposed CCB uses vector quantization (VQ) technology [38], which can reduce the complexity of channel estimation compared with traditional design methods. Firstly, the wireless channel parameter *H* is extracted, where

(7) $$H=\left(\begin{array}{cccc} h_{11} & h_{12} & \cdots & h_{1N}\\ h_{21} & h_{22} & \cdots & h_{2N}\\ & & \ddots & \\ h_{M1} & h_{M1} & \cdots & h_{MN} \end{array}\right).$$

Specifically, the parameters of *H* are determined by various parameters and SNR values in the SR channel. This process enables the dynamic adjustment of code-book parameters in response to changing channel conditions, thereby facilitating adaptation to different noise environments. This ensures that the model maintains optimal performance under varying signal conditions.

Then the extracted channel characteristic parameters are quantified to generate the feature vector *v*, that is,

(8) $$v=f\left(H\right)=\sigma \left(W_{f}H+B\right),$$

where σ is a nonlinear activation function, $W_{f}$ is the weight matrix, and *B* is the bias vector. Theoretically, different channel matrices may be mapped to the same feature vector, so the denoising parameters optimized for $H_{1}$ may not be effective for $H_{2}$. Therefore, when we perform feature mapping, we use multi-level feature vectors to represent channel information more comprehensively, which can reduce the probability that different channel matrices are mapped to the same feature vector.

Finally, the CCB is constructed by VQ technology, and the channel feature vector is mapped to code-book index *c* for

(9) $$c=Q\left(v\right)=\arg\min_{c_{i}\in C}\left\|v-c_{i}\right\|,$$

where *v* is the feature vector, $c_{i}$ is the codeword in the code-book, and *C* is a set of code-books.

### 3.2. Channel Code-Book MappingNet

Faced with the complex satellite–ground environments, a single channel parameter expression cannot accurately describe the current actual situation. Therefore, to improve the performance and reliability of communication systems, we propose the Channel Code-Book MappingNet. The network consists of two main modules: Channel Code-Book and Denoising Parameter. Channel Code-Book is used to store different channel parameters, and Denoising Parameter is used to store the denoising model parameters under different channel conditions. The channel state mapping network realizes the accurate description and optimization of the channel environment by extracting and mapping the channel state information to the corresponding denoising parameters.

### 3.3. Training Algorithm

To establish the channel parameter mapping relationship under different states of the SR channel, we first record different channel parameters. The channel parameters consist of four parts: $b_{0}$, *m*, Ω, and SNR. Then, we train the corresponding denoising parameters through the recorded channel parameters. In this step, the goal of this process is to minimize the difference between $x^{\prime \prime }$ and x, that is, $\left(\theta^{*}\right)=\arg\min_{\theta }\left[d\left(x,x^{\prime \prime }\right)\right]$. By changing the parameters of the current training channel model, the CCB MappingNet can obtain and store the channel parameters of different environments and the denoising model parameters mapped to them. The training procedure is outlined in Algorithm 2.

By this method, image transmission in different channel environments can be realized through a set of systems in the satellite–ground scene, which can greatly reduce the resource occupation of the system under the premise of ensuring the quality of data recovery.

## 4. Proposed CCB-Guided Adaptive Channel Denoising Mechanism

To adapt to the complex channel conditions under satellite–ground conditions, improve transmission quality, and reconstruct the image fidelity, we propose a denoiser module and a model parameter selection mechanism. The denoiser module dynamically adjusts the parameters and configuration of the model by modeling and storing different channel environment parameters to best adapt to different channel qualities. The model parameter selection mechanism judges the current actual channel parameters through the pilot signal and dynamically selects the appropriate denoiser model parameters to achieve the best transmission performance under different channel and SNR conditions, thereby improving the robustness and reliability of the system.

### 4.1. Architecture of Adaptive Channel Denoising Model

We propose an adaptive channel dependence mechanism, which enables the end-to-end image transmission system to automatically adapt to changes in channel state without manual intervention. As the denoiser module, ACDM is designed to enhance the stability and performance of the system.

**Figure 3:**
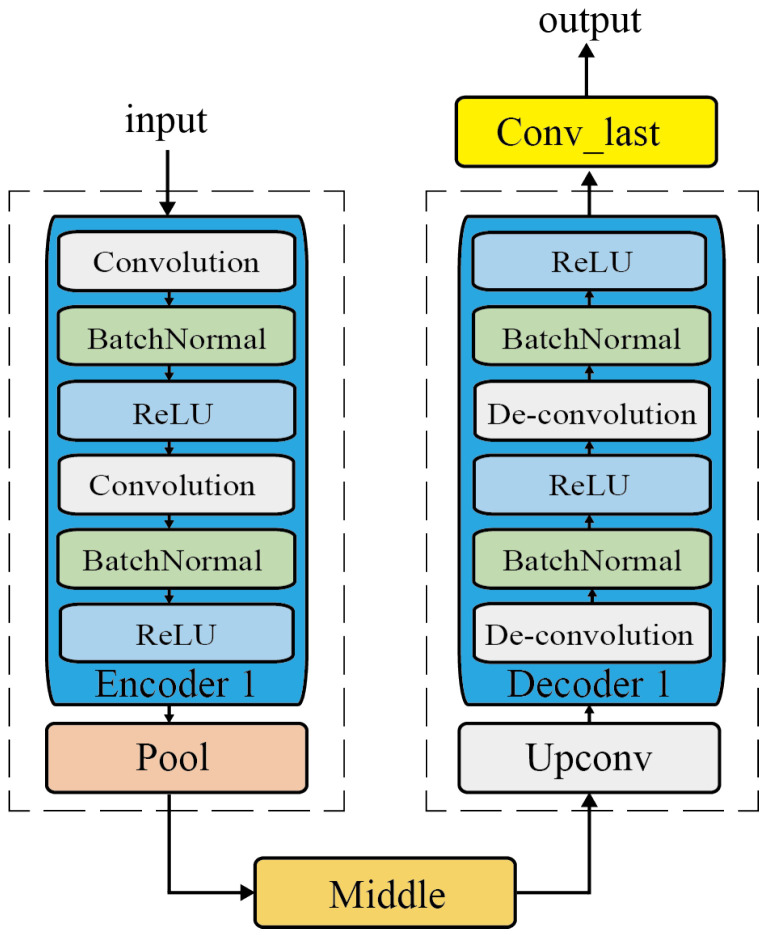
The architecture of ACDM.

As shown in Figure 3, ACDM consists of a UNet architecture, including the encoder1 and the corresponding decoder1, an intermediate layer, and a final output layer. The reason for using the UNet model is that the model uses skip connections to directly transfer the high-resolution feature maps extracted from each encoder layer to the corresponding decoder layer. Skip connection provides a direct path from the early layer of the network to the higher layer for high-resolution functions. When an image passes through multiple convolutional layers, it will undergo multiple transformations, which may lead to information loss. Skipping the connection helps to bypass these transformations, allowing the original high-resolution information to be retained and reintroduced into the later stages of the network. Therefore, by preserving high-frequency components and fine details, skipping the connection ensures that the denoised image retains clarity. In this way, not only is the global semantic information retained during the upsampling process, but also the rich spatial details are retained. Then, since the denoiser module only needs to recover the fading of semantic features in the channel without restoring the entire image, we reduced the number of layers in the model to two and the number of channels from 64 to 2, greatly increasing the system’s operating efficiency while ensuring the completion of the task.

In this architecture, encoder1 converts the input data *x* into the probability distribution parameter of the latent variable *z*, that is,

(10) $$q\left(z|x\right)=N(z;\mu \left(x\right),\sigma^{2}\left(x\right)),$$

where μ(x) and $\sigma^{2}\left(x\right)$ are the mean and variance of latent variables, respectively. These parameters are generated by encoder1 to help the model sample reasonably in the data space.

Correspondingly, decoder1 generates the distribution of the reconstructed data *x* through the latent variable *z* for

(11) $$p\left(x|z\right)=N(x;f\left(z\right),\sigma^{2}),$$

where f(z) is the mean function generated by decoder1 and $\sigma^{2}$ is the variance. By maximizing p(z), the parameters of decoder1 can be optimized to make the reconstructed image closer to the original input.

We adopt Kullback–Leibler (KL) divergence to measure the difference between the posterior distribution q(z|x) of the latent variable and the prior distribution p(z), with

(12) $$L_{\mathrm{KL}}=D_{\mathrm{KL}}\left(q\left(z|x\right)\|p\left(z\right)\right),$$

where $D_{\mathrm{KL}}\left(q\left(z|x\right)\|p\left(z\right)\right)=\int q\left(z|x\right)\log\frac{q(z|x)}{p(z)}dz$. Specifically, the prior distribution p(z) is derived from the statistical characteristics of the actual data, effectively capturing the true distribution properties of the data. By leveraging this prior and the obtained posterior knowledge, the KL divergence is calculated using integral methods. This approach ensures that the model accurately reflects the underlying data distribution and optimizes its performance in various applications.

### 4.2. Training Algorithm for Channel DenoisingNet

The training of Channel DenoisingNet mainly consists of two parts: encoder1 and decoder1. The training algorithm is shown in Algorithm 3. encoder1 consists of two convolutional layers that extract information from the input features, and each layer applies convolutional filters to capture spatial hierarchies of patterns, such as edges and textures. The mapping procedure is

(13) $$f_{conv}\left(x^{\prime }\right)=W_{conv}*x^{\prime }+b_{conv},$$

where $W_{conv}$ and $b_{conv}$ are the convolution kernel and bias term, respectively. * represents the convolution operation. After convolution, a pooling operation reduces the spatial dimensions (height and width) of the feature maps, retaining essential information while downsampling the data. This pooling increases the receptive field, allowing the network to understand larger context from the image.

The structure of the decoder1 mirror, i.e., encoder1, realizes the precise positioning of the features. decoder1 reconstructs the segmented image according to the coding features, which contain two convolutional layers and an upsampling layer. The upsampling layer consists of a series of upsampling operations (such as deconvolution or transposed convolution) and convolution operations, which are used to gradually increase the size and number of channels of the feature map. This can gradually restore the resolution and retain more details, and the mapping procedure is

(14) $$x^{\prime \prime }=f_{deconv}\left(a\right)=W_{deconv}*a+b_{deconv},$$

where $W_{deconv}$ and $b_{deconv}$ are the deconvolution kernel and bias term, respectively, and a is the output of the previous stage.

Through the training algorithm for Channel Code-Book MappingNet in Section 3, the model can obtain the different values of W and b based on $\theta^{*}$. By choosing the values of W and b, the denoiser can adjust the parameters according to different channel states to adapt to dynamic channels.

### 4.3. Denoiser Parameter Selection Mechanism

Algorithm 4 outlines the image processing process. First, the input image is processed to feature vector by the encoder. The transmitter will then check if it is the first transmission. If it is, the transmitter sends the polit feature vector $x_{0}$, which is generated from a fixed image; the receiver receives $x_{0}^{\prime }$ after the wireless channel and compares it with the pre-stored fading vector $x_{n}^{\prime }$ (n=1,2,…,N) to choose the closest vector $x_{\min}^{\prime }$. When it obtains $x_{\min}^{\prime }$, the denosier will set the denoiser parameter θ, converted by $x_{\min}^{\prime }$. After completing the above steps, the subsequent transmission image will pass the denoiser using this parameter model and enter the decoder for image reconstruction.

## 5. Simulation Results and Analysis

### 5.1. Dataset

We utilize the CIFAR10 dataset [39] to assess the transmission quality of the CCB model and the DIV2K dataset [40] for image visualization training and verification. The CIFAR10 dataset, a well-known resource for universal object recognition in computer vision, consists of 60,000 32 × 32 RGB color images across 10 categories. Of these, 50,000 images are allocated to the training set, and 10,000 images to the testing set. The DIV2K dataset comprises 800 high-resolution training images and 100 high-resolution verification images, each characterized by exceptional clarity, making it ideal for training and evaluating super-resolution algorithms.

### 5.2. Baseline Schemes

#### 5.2.1. Deep JSCC

Deep JSCC [41] is a scheme that leverages deep learning to jointly optimize source coding and channel coding. Unlike traditional communication systems, which typically treat these two processes separately, Deep JSCC employs convolutional neural networks (CNNs) for end-to-end learning. This approach enables the simultaneous optimization of both processes, leading to improved communication efficiency and reliability. However, it should be noted that this method may not perform well in rapidly changing environments.

#### 5.2.2. ASCN

ASCN [33] is a scheme utilizing a single deep neural network (DNN) that adaptively adjusts the transmission rate at three levels of SR fading. This adjustment is based on the input image features and time-varying channel state information. Across these SR fading levels, ASCN uses pilots to obtain channel state information and performs semantic matching according to image features, resulting in a significant improvement in image reconstruction quality compared with existing schemes.

#### 5.2.3. WITT

WITT [42] is a method that utilizes the swin transformer as its backbone to optimize image transmission, taking into account the impact of wireless channels. The authors propose a spatial modulation module that scales the latent representations based on channel state information. This enhances the model’s ability to manage various channel conditions effectively. Furthermore, the scheme can be extended to a generalized version, improving its adaptability to different channel conditions and rate configurations.

### 5.3. Performance Metrics

#### 5.3.1. PSNR

Peak Signal-to-Noise Ratio (PSNR) is an index used to measure the quality of images or signals. It is usually used to evaluate the similarity between an image and the original image, especially in the field of image compression and reconstruction. The higher the PSNR value, the higher the similarity between the two images, the better the quality. The calculation formula is

(15) $$PSNR=10\cdot log_{10}\left(\frac{max^{2}}{MSE}\right),$$

where max represents the maximum possible value of the pixel value, usually 255 in image processing, and MSE represents the mean square error.

#### 5.3.2. SSIM

Structural Similarity Index Measure (SSIM) is a perceptual metric that aims to approximate human visual perception when comparing the quality of digital images. SSIM is widely used in the field of image processing and computer vision [42,43,44]. It is a more comprehensive and perceptually consistent image quality assessment method. The SSIM value ranges between 0 and 1, where a value closer to 1 indicates greater similarity between the two images being compared. The calculation formula is

(16) $$SSIM\left(x,y\right)=\frac{\left(2\mu_{x}\mu_{y}+c_{1}\right)\left(2\sigma_{xy}+c_{2}\right)}{\left(\mu_{x}^{2}+\mu_{y}^{2}+c_{1}\right)\left(\sigma_{x}^{2}+\sigma_{y}^{2}+c_{2}\right)},$$

where $\mu_{x}$ and $\mu_{y}$ are the average values of *x* and *y*, $\sigma_{x}^{2}$ and $\sigma_{y}^{2}$ are the variances of *x* and *y*, and $\sigma_{xy}$ is the covariance of *x* and *y*. $c_{1}$ and $c_{2}$ are constants used to maintain stability, usually set to $c_{1}={\left(k_{1}L\right)}^{2}$ and $c_{2}={\left(k_{2}L\right)}^{2}$, where *L* is the dynamic range of pixel values, and $k_{1}$ and $k_{2}$ are default values that usually take $k_{1}$ = 0.01 and $k_{2}$ = 0.03.

### 5.4. Simulation Parameters

Table 1 presents the model structure and the output size for each layer. In the entire model, we employ the Gaussian Error Linear Unit (GELU) as the activation function. GELU is a nonlinear activation function that integrates the properties of the Gaussian distribution with a linear function. This combination enables the function to capture complex data patterns, enhancing the model’s ability to understand intricate details within images and effectively capture the nonlinear characteristics of the data. For the CIFAR10 dataset, we use two stages: [N1,N2] = [2, 4], [C1,C2] = [128, 256], with the window size set to 2. For the DIV2K dataset, we utilize four stages: [N1,N2,N3,N4] = [2, 2, 6, 2], [C1,C2,C3,C4] = [128, 192, 256, 320], with the window size set to 8. The model structure of baseline is shown in Table 2.

We employ the Adam optimizer with a learning rate of 0.0001. The batch sizes of the CIFAR10 dataset and the DIV2K dataset are set to 128 and 4, respectively. The CCB model is trained in the SR channel with SNR from −5 dB to 20 dB, and the channel parameters are shown in Table 3. To initialize the codebook entries, we first utilize the Gaussian distribution to randomly generate initial values. After completing the training of a channel model, the pre-trained model parameters are subsequently employed to initialize and train other channel models. All these parameters are implemented in the PyTorch architecture, utilizing the NVIDIA A10 GPU.

### 5.5. Results Analysis

#### 5.5.1. PSNR Performance

**Figure 4:**
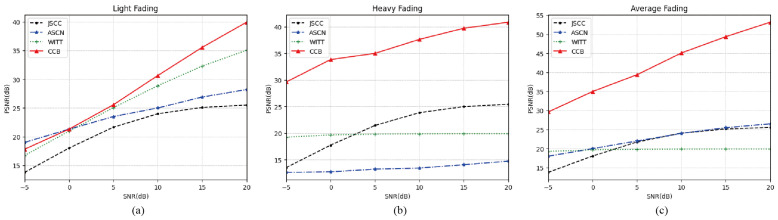
(**a**) PSNR performance over the light fading channel. (**b**) PSNR performance over the heavy fading channel. (**c**) PSNR performance over the average fading channel.

Figure 4 provides a detailed depiction of the PSNR performance of the proposed CCB model across various SNR constraints in SR fading channels with differing parameters. The model demonstrates strong adaptability to varying signal-to-noise ratios and channel conditions, with performance that is equivalent to or even surpasses that of JSCC and WITT. Particularly, in heavy environments, where channel noise significantly impacts the system, the challenge to decode information semantics becomes greater. This may be attributed to the utilization of neural networks for encoding and decoding, avoiding the introduction of additional redundancies that undermine other schemes. Due to the targeted training of the channel parameters specific to the scene, our scheme exhibits a performance advantage of more than 10 dB across all SNRs when compared to other models. The results indicate that, as a versatile model, our CCB achieves satisfactory SNR adaptation in complex satellite–ground channel environments, with negligible performance loss.

#### 5.5.2. SSIM Performance

**Figure 5:**
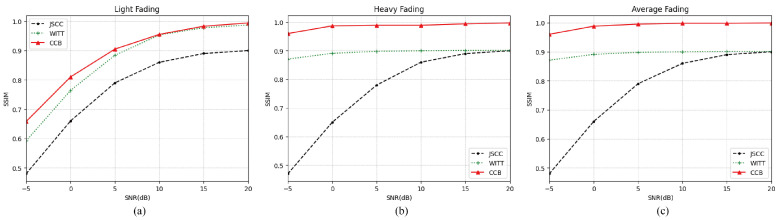
(**a**) SSIM performance over the light fading channel. (**b**) SSIM performance over the heavy fading channel. (**c**) SSIM performance over the average fading channel.

Figure 5 illustrates the SSIM performance of the proposed CCB model under varying SNR constraints in SR fading channels with different parameters. Utilizing an encoder based on a swin transformer, our CCB model achieves a substantial recovery of the original image, with SSIM values approaching 1 at high SNR. In contrast, the Deep JSCC model, which relies on complex JSCC strategies, struggles to cope with high noise interference, leading to increased decoding errors and poor performance in the low-SNR environment. Moreover, our model demonstrates strong adaptability to different channel conditions, achieving performance comparable to or even exceeding that of the reference model. The results indicate that our CCB model maintains robust performance across both low- and high-SNR conditions, particularly excelling in high-SNR scenarios, where it effectively reconstructs the information conveyed by the original image.

#### 5.5.3. Visualization Performance

To compare the advantages and disadvantages of various schemes more intuitively, we reconstruct high-definition images on the DVI2K dataset in a visual way.

**Figure 6:**
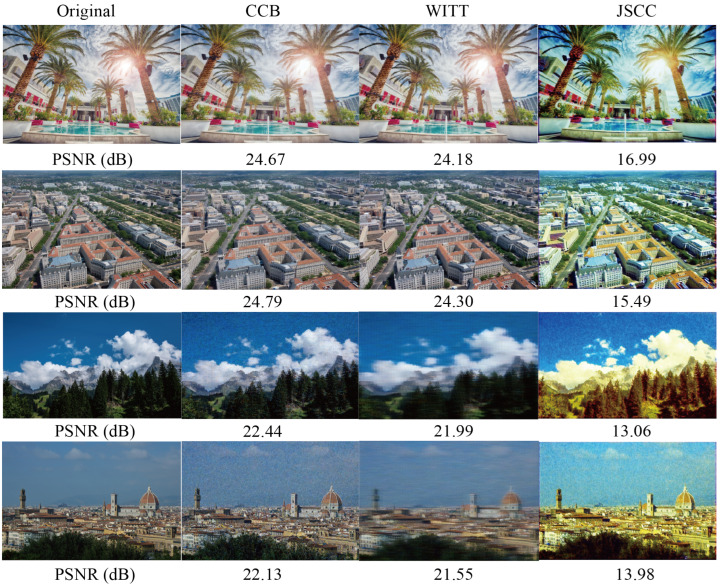
The first two lines are visual comparison examples under light fading with SNR = 20 dB. The last two lines are visual comparison examples under light fading with SNR = 5 dB. The first column to the fourth column show the original image and the reconstructed image under different transmission schemes.

Figure 6 illustrates the visualization results obtained from various image transmission schemes under different SNR conditions. Under high-SNR conditions, both our proposed scheme and the WITT scheme effectively restore the original image. In contrast, the JSCC scheme exhibits an obvious color cast issue, attributed to the limitations of the CNN model in high-precision image processing. Under low-SNR conditions, despite the presence of numerous noise points, our proposed scheme outperforms other schemes in terms of image restoration accuracy due to its superior anti-noise capabilities. These results demonstrate that our proposed CCB transmission scheme provides better visual quality compared to other schemes.

#### 5.5.4. Robustness Performance

Due to the complex satellite–ground transmission environment, it is impossible to take all channel conditions into account during model training. Therefore, it is necessary to test the robustness of the model to ensure that the proposed model can still work stably in non-training scenarios.

**Figure 7:**
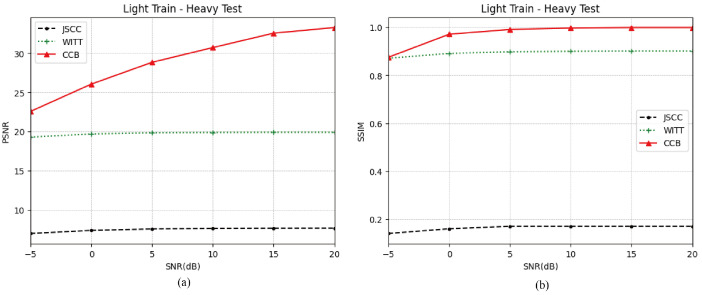
(**a**) PSNR performance under different training scenarios and test scenarios. (**b**) SSIM performance under different training scenarios and test scenarios.

Figure 7 illustrates the performance of the proposed CCB model under different testing and training environments. The training scenario involves a light fading channel, while the test scenario involves a heavy fading channel. The diagram indicates that the JSCC model, which employs a CNN mechanism, has a relatively simple architecture and thus relies heavily on the training environment. Consequently, when the test environment differs, its PSNR falls significantly below 10 dB, and its SSIM drops below 0.2, rendering image transmission nearly impossible. The introduction of a pilot mechanism in our proposed CCB model allows it to select model parameters that are most closely aligned with the actual test scenario. As a result, it can improve by more than 2 dB compared to the WITT model at low SNR. These results demonstrate that the CCB model performs well, even in the absence of corresponding parameters for the current test environment.

**Figure 8:**
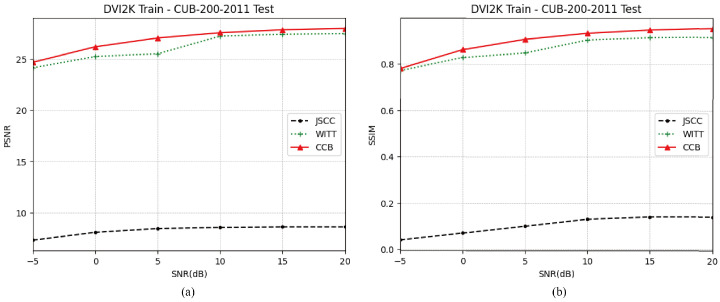
(**a**) PSNR performance under different training dataset and test dataset. (**b**) SSIM performance under different training dataset and test dataset.

Figure 8 illustrates the performance of the proposed CCB model under different training and test datasets, simulating various satellite transmission image tasks. The training dataset used is DIV2K, while the test dataset is CUB-200-2011 [45]. The graph shows that the performance of the proposed CCB model is comparable to the WITT model, with a PSNR exceeding 25 dB. This is because, in high-SNR environments, noise interference on image restoration is minimal. As a result, the quality of image restoration predominantly relies on the inherent capabilities of the encoder and decoder. Both CCB and WITT models utilize an architecture based on the swin transformer, which captures more semantic information. In contrast, the Deep JSCC model, employing a simpler CNN architecture, heavily relies on the features within the training dataset. When there is a significant disparity between the test and training datasets, the JSCC model struggles to adapt to new data features, resulting in a substantial performance drop. Consequently, the SSIM of the JSCC model in the new test dataset falls below 0.2, rendering it practically unusable.

These results highlight the superior adaptability and robustness of the proposed CCB model in handling diverse and complex test scenarios compared to other models.

#### 5.5.5. Latency and Complexity Performance

We use inference time to characterize the latency and complexity of the model across various stages. Table 4 provides a comparative analysis of the inference time among different model schemes when processing the test set. As seen in the table, the Deep JSCC model exhibits the shortest delay due to its reliance solely on the CNN architecture; however, this also results in suboptimal image restoration quality. The latencies associated with the CCB and WITT models are similar because both employ comparable codec architectures. Nonetheless, the WITT model experiences longer delays due to the necessity of channel estimation through feedback. Consequently, our proposed CCB model preserves transmission quality without increasing model complexity.

## 6. Conclusions

Satellite–ground communication faces significant challenges in data transmission efficiency due to the dynamic nature of satellite communication channels. In this paper, we have presented a CCB architecture for wireless image transmission in satellite–ground scenarios, which demonstrates the ability to flexibly adapt to different channel states in complex channel environments. Firstly, we construct a well-designed CCB framework based on the emerging swin transformer backbone network, which outperforms the traditional CNN-based neural JSCC. Then, we design a MappingNet to map the environmental parameters to the corresponding decoding parameters. Through joint training under different satellite–ground transmission channels, we construct a CCB-based denoiser parameter library and determine the model parameters of the denoiser by sending specific pilots, so as to realize adaptive adjustment of different channels and noises without changing the state of the encoder. The experimental results show that, compared to the CNN-based JSCC system and the ordinary swin transformer system WITT, our CCB model can achieve better performance, adapt to more channel environments, and have stronger robustness, especially under high SNR conditions.

In future work, to better adapt to real satellite–ground image communication scenarios, we plan to incorporate a broader range of channel environments for training and testing. This will include the use of a frequency-selective fading tapped delay line (TDL) channel model and Doppler frequency-shift satellite channels. By diversifying the channel conditions, we aim to enhance the robustness and effectiveness of our communication model across different real-world environments.In addition, to more accurately evaluate the performance of image transmission and semantic loss in downstream tasks (such as object detection or classification), we plan to adopt more comprehensive evaluation metrics, such as accuracy and mean average precision (mAP).

## Figures and Tables

**Table 1:** Model structure.

	Layers	Output Size
Encoder	PatchEmbed	256
	SwinTransformerBlock × 2	128
	SwinTransformerBlock × 4	256
	LayerNorm	256
	Linear	96
Denoiser	Encoder1	(1, 128, 64, 96)
	Middle	(4, 64, 32, 48)
	Decoder1	(2, 128, 64, 96)
	Conv_last	(1, 128, 64, 96)
Decoder	SwinTransformerBlock × 4	256
	SwinTransformerBlock × 2	128
	PatchReverseMerging	128
	Linear	256

**Table 2:** Baseline model structure.

Model	Type	Layers
Deep JSCC	Encoder	(Conv + PReLU) × 5
	Decoder	(Transconv + PReLU) × 5
		Transconv + sigmoid
ASCN	Encoder	(Conv + ReLU) × 3
		(ResNet + SNR Adaptive) × 2
		SR Channel ModNet
	Decoder	SR Channel ModNet
		(ResNet + SNR Adaptive) × 2
		(Transconv + ReLU) × 2
		Conv + sigmoid
WITT	Encoder	Patch Embedding + Swin Transformer Block
		(Patch Merging + Swin Transformer Block) × 3
		Channel ModNet
	Decoder	Channel ModNet
		(Patch Division + Swin Transformer Block) × 4

**Table 3:** SR channel parameters.

Type/Parameter	$b_{0}$	*m*	Ω
Light	0.158	19.4	1.29
Heavy	0.063	0.739	$8.79\times 10^{-4}$
Average	0.126	10.1	0.835

**Table 4:** Model inference time.

Model	CCB	WITT	Deep JSCC
Inference Time (s)	3.7689	4.2914	2.8360

## Data Availability

Data will be made available on request.
